# Structure of tetra­kis­(penta­fluoro­phenoxido)bis­(tetra­hydro­furan)­titanium(IV)

**DOI:** 10.1107/S2056989026007024

**Published:** 2026-07-16

**Authors:** William G. Van Der Sluys

**Affiliations:** a Department of Chemistry The Pennsylvania State University Commonwealth College at Altoona Altoona Pennsylvania 16601 USA; Harvard University, USA

**Keywords:** alkoxides, crystal structure, fluoro­alkoxides, ionization isomer, coordinating solvent, Ziegler–Natta polymerization catalyst

## Abstract

Titanium(IV) chloride was reacted with a slight excess of barium penta­fluoro­phenoxide in tetra­hydro­furan (THF) solution. After filtration and solvent evaporation, the resulting orange crystalline material was obtained. The product was recrystallized by preparing a concentrated solution in THF at room tem­per­a­ture, followed by cooling to 253 K. The supernatant solution was deca­nted using a pipet, and the crystals were dried by allowing the solvent to evaporate in the atmosphere of a glovebox. It was possible to obtain good-quality X-ray data at 100 K on a crystal, namely, tetra­kis­(penta­fluoro­phenoxido-κ*O*)bis­(tetra­hy­dro­furan-κ*O*)titanium(IV), [Ti(C_6_F_5_O)_4_(C_4_H_8_O)_2_], having monoclinic (*P*2_1_/*n*) symmetry. This com­pound adopts a solid-state structure in which the titanium(IV) ions have a distorted octa­hedral coordination environment with two coordinated THF mol­ecules occupying *cis* positions.

## Abstract 2

The crystal structure of the tetra­kis­(penta­fluoro­phen­oxy)bis­(tetra­hydro­furan)­titanium(IV) com­plex is described, in which the titanium(IV) ion adopts a distorted octa­hedral coordination geometry. The coordinated tetra­hydro­furan ligands adopt a *cis* configuration.

## 1. Chemical context

Early transition-metal alkoxides have been of inter­est for applications ranging from organic synthesis and catalysis to the preparation of metal oxide materials *via* the sol-gel process (Bradley, 1959 ▸; Bradley *et al.*, 1978 ▸; Bradley *et al.* 2001 ▸). Earlier work by the author of this article assumed the use of fluorinated alkoxides would provide enhanced Lewis acidity, while maintaining steric and stereochemical control over Ziegler–Natta polymerization-type processes. These publications described the partial substitution of alkyl alkoxides by fluoro­alkoxides in alcoholysis-type reactions (Campbell *et al.*, 1994 ▸; Fisher *et al.*, 1993 ▸).
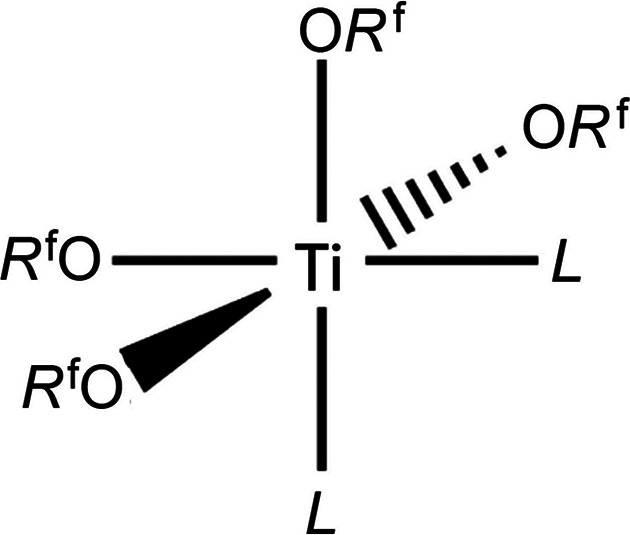


In addition, the structure of an aceto­nitrile adduct of tetra­kis­fluoro­alkoxide titanium(IV), Ti[OCH(CF_3_)_2_]_4_(N≡CCH_3_)_2_, was described, in which the coordinated solvent mol­ecules act as Lewis bases in *cis* positions by way of nitro­gen lone pairs. The coordination geometry of the titanium(IV) ion was best described as being a distorted octa­hedral structure. It was tempting to assume that this structure was typical for the disolvates of the tetra­kis­fluoralkoxide titanium(IV) com­plexes.

However, it was not possible to obtain suitable quality X-ray data for the analogous tetra­hydro­furan (THF) adduct due to the significantly low melting point. However, the data that were obtained seemed to suggest that the THF adduct was an ionization isomer in the solid state com­prised of a Ti(OPr^f^)_2_THF_4_^2+^ cation and a Ti(OPr^f^)_6_^2−^ anion (OPr^f^ is hexa­fluoro­isopropoxide). Ionization isomers of metal com­plexes are known (Barbier *et al.*, 1972 ▸; Tebbe & Muetterties, 1967 ▸; Kamata *et al.*, 2012 ▸; Giesbrecht *et al.*, 2004 ▸; Xie *et al.*, 1996 ▸; Niemeyer, 2001 ▸), and it was assumed that the enhanced electron-withdrawing ability of the fluoro­alkoxides as com­pared with the electron-donor properties of alkyl alkoxides would have a preference for such an ionization isomer in a polar solvent such as THF. Malinowski & Koteras (2026 ▸) recently described the crystal structure of [Ti{OC(CF_3_)_3_}_2_(N≡CCH_3_)_4_][Al{OC(CF_3_)_3_}_4_], with the perfluoro­alkoxide ligands coordinated to the titanium(III) cation exhibiting a significant amount of disorder.

Some recent efforts to obtain X-ray-quality crystals of the Ti[OCH(CF_3_)_2_]_4_THF_2_ com­plex, which was prepared by way of a metathesis reaction between titanium(IV) chloride and sodium hexa­fluoro­isopropoxide in THF as a solvent, have been published (Van Der Sluys, 2024 ▸), but were again not entirely successful. However, it was possible to obtain the structure of a slightly higher melting minor product, in which only three chlorides had been substituted by fluoro­alkoxides, producing TiCl(OPr^f^)_3_THF_2_. In the present article, the X-ray crystal structure of the related penta­fluoro­phenoxide com­plex, Ti(OC_6_F_5_)_4_THF_2_ (com­plex **1**), is reported.

## 2. Structural commentary

**Figure 1:**
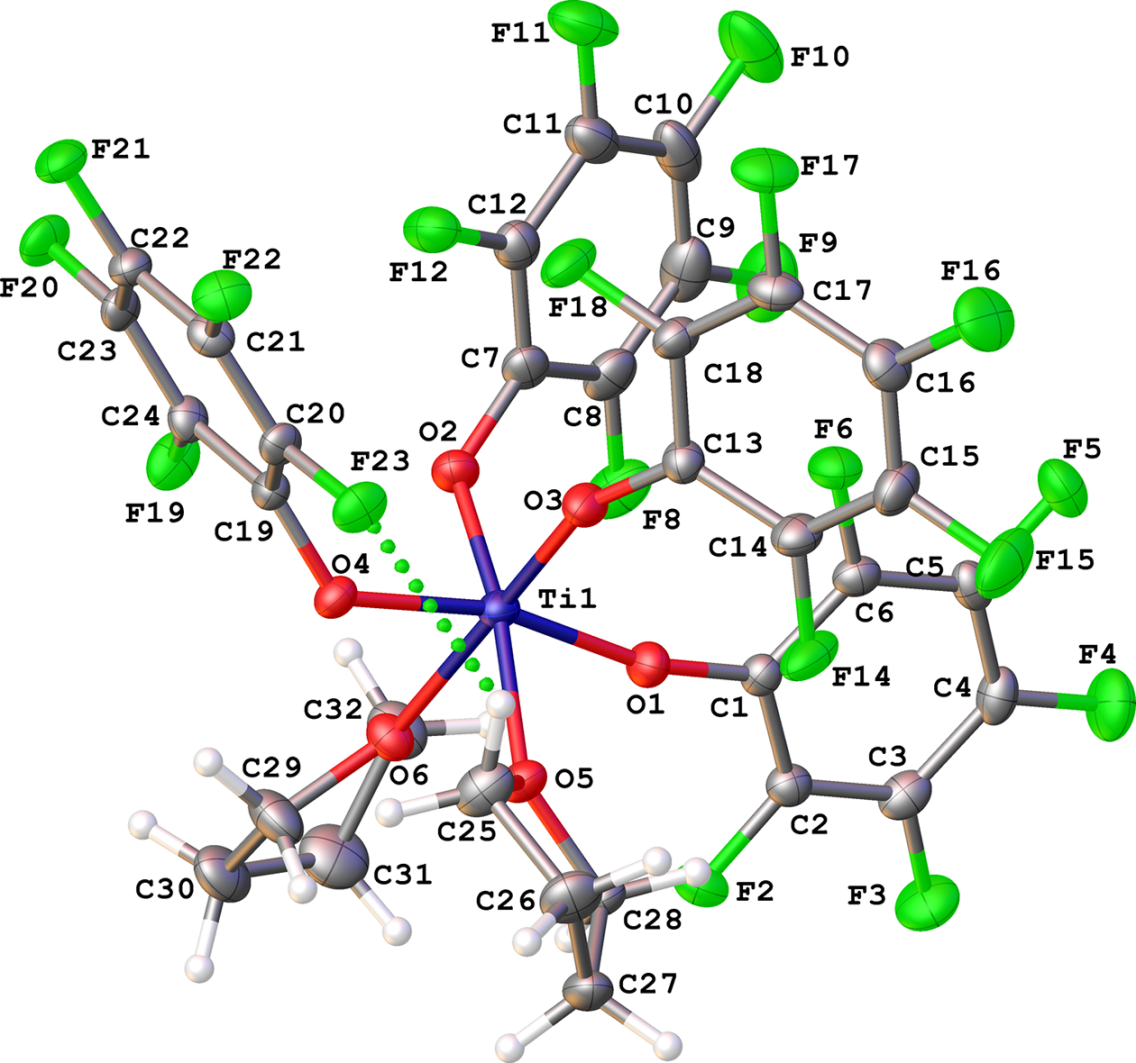
The mol­ecular structure of **1**, showing the atom-numbering scheme used. Displacement ellipsoids are drawn at the 50% probability level.

The mol­ecular structure of **1** shown in Fig. 1 ▸ emphasizes the nearly octa­hedral coordination geometry of the Ti atom, with the THF ligands adopting a *cis* configuration. Guzei & Winter (1997 ▸) have presented arguments concerning the factors that affect the formation of *cis versus trans* isomers in TiCl_4_*L*_2_ com­plexes containing pyrazole ligands. Their arguments sug­gest that π-donor ligands greatly influence the stability of the *cis versus trans* isomers. Alkoxide ligands are generally con­sidered better π-donor ligands than chloride ligands, but presumably the fluoro­phenoxide ligands somewhat attenuate these properties due to the electron-withdrawing nature of the F atoms and the potential delocalization of the oxygen lone pairs into the benzene π-ring system. Fractional coordinates and other crystallographic data can be found in the supporting information.

The coordination geometry of **1** is best described as distorted octa­hedral. The fluoro­phenoxide Ti—O bond lengths [average 1.8691 (19) Å] are com­parable to those found in other fluoro­phenoxide com­pounds whose structures have been published previously (Campbell *et al.*, 1994 ▸; Fisher *et al.*, 1993 ▸). The Ti—O bond lengths for the coordinated THF ligands [average 2.1195 (18) Å] are significantly longer than the phenoxide bond lengths, com­parable to those previously observed (Van Der Sluys, 2024 ▸), and consistent with the weaker polar coordinate bonds associated with the neutral ether ligands as com­pared with the phenoxide ligands.

## 3. Supra­molecular features

**Figure 2:**
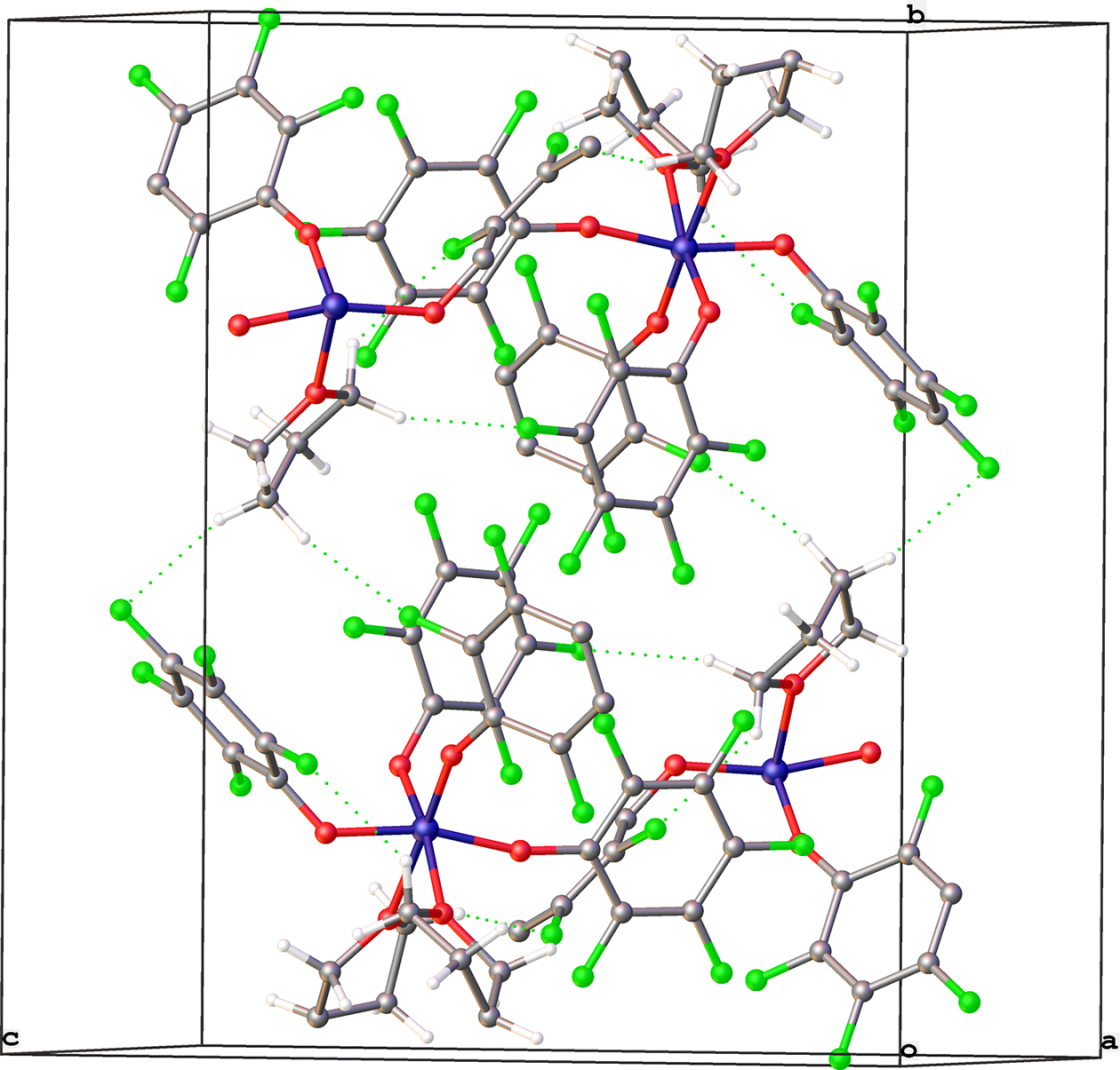
Packing diagram for the unit cell of **1**.

Two of the fluoro­phenoxides in **1** have significantly more obtuse Ti—O—C bond angles and shorter Ti—O bond lengths than the other two fluorophenoxides. Specifically, the bond lengths and angles for the fluoro­phenoxides that are *trans* to the THF ligands are Ti1—O2 and Ti1—O3, which are 1.8777 (18) and 1.8370 (18) Å, respectively, while the Ti1—O2—C7 and Ti1—O3—C13 angles are 131.35 (17) and 158.45 (17)°, respectively. Alternatively, the fluoro­phenoxide ligands that are *trans* to each other, Ti1—O1 and Ti1—O4, have bond lengths of 1.8548 (19) and 1.9068 (19) Å, respectively, and Ti1—O1—C1 and Ti1—O4—C1 angles of 166.99 (17) and 129.21 (16)°, respectively. These bond angles do not seem to correlate well with anti­cipated *trans*-influence effects associated with phenoxide ligands being *trans* to a phenoxide *versus* a THF ligand. The larger Ti—O—C bond angles and shorter Ti—O bond lengths could be inter­preted as resulting from more significant π bonding inter­actions of the oxygen lone-pair inter­actions with the empty *t*_2*g*_ (*d_xy_*, *d_xz_* and *d_yz_*) set of *d* orbitals of the titanium(IV) ion in an octa­hedral ligand field, which has a 3*d*^0^ electronic configuration. These variations do not seem to correlate with significant changes in the C—O bond lengths, which might be anti­cipated from changes in the bonding delocalization inter­actions of the benzene and the oxygen lone pairs. These variations in bond angles and lengths may simply be a result of crystal packing forces that influence the orientations of the benzene rings, as shown in the unit-cell packing diagram in Fig. 2 ▸.

## 4. Synthesis and crystallization

The reaction of penta­fluoro­phenol with various alkoxides of titanium(IV) resulted in incom­plete substitution of the alkyl alkoxide ligands (Campbell *et al.*, 1994 ▸). Mazdiyasni *et al.* (1971 ▸) described the synthesis of a series of hexa­fluoro­isopropoxide Group IV element com­pounds by way of a metathesis approach, in which the metal chlorides were reacted with four equivalents of the sodium fluoro­alkoxides, with the corresponding liquid fluoro­alcohols as the solvent. The desired com­pounds were obtained by fractional distillation and could be recrystallized from various organic solvents, including diethyl ether and THF. It was hoped that a similar metathesis approach could be used to prepare the corresponding titanium(IV) penta­fluoro­phenoxide com­pounds, but since penta­fluoro­phenol is a solid at room tem­per­a­ture, THF was used as the solvent. A potential com­plicating factor in the reaction with greater than four equivalents of alkoxide is the potential formation of anionic species, such as [Ti(O*R*)_5_]^−^ (Boyle *et al.*, 1999 ▸; Chandler *et al.*, 1993 ▸) and [Ti(O*R*)_6_]^2−^ (Day *et al.*, 1995 ▸), which have been shown to be useful in controlling sol-gel production of titanium oxide solid-state materials.

Reaction of titanium(IV) chloride with greater than two equivalents of barium penta­fluoro­phenoxide, which was pre­pared *in situ* from barium metal and penta­fluoro­phenol, resulted in the rapid formation of an orange solution and a white precipitate. The solution was stirred at room tem­per­a­ture for 12 h, after which the solution was filtered, and the volume of the solution was reduced *in vacuo*. Cooling the solution to 253 K resulted in the formation of orange crystals, which were characterized by SEM-XPS and IR spectroscopy (see supporting information). The crystalline product has been formulated as Ti(OC_6_F_5_)_4_THF_2_. It was somewhat disappointing that this reaction did not appear to produce a double alkoxide com­plex containing barium and titanium(IV) ions similar to the BaTi(OC_6_H_5_)_6_·2DMF com­pound (Day *et al.*, 1995 ▸), which was used to prepare barium titanate by way of the sol-gel process.

Liquid TiCl_4_ and solid TiCl_4_THF_2_ were purchased from Aldrich and used as received. All synthetic procedures were carried out using standard Schlenk techniques or in a purified nitro­gen atmosphere using a Braun UNIlab glovebox. Sol­vents were purified by distillation from a sodium benzo­phenone ketal solution and stored in glass containers with solvent seal fittings. IR spectra were recorded as KBr pellets, by grinding small portions of the sample with dried potassium bromide using an agate mortar and pestle in the glovebox. Fourier transform IR (FT–IR) spectra (Fig. S1 in the sup­porting information) were recorded on a ThermoScientific Nicolet iS10 FT–IR spectrometer. Residual gaseous carbon dioxide asymmetric vibrations were sometimes observed in the 2400 cm^−1^ region, due to incom­plete background subtraction, and calibration was checked regularly using a film of polystyrene. Scanning electron micrograph (SEM) images and X-ray photoelectron spectroscopy (XPS) for com­po­sitional analysis (Fig. S2) were obtained by the Materials Characterization Lab of the Materials Research Institute at the PSU University Park campus.

## 5. Refinement

Crystal data collection and structure refinement details are summarized in Table 1 ▸. The positions of the H atoms were initially determined by geometry and were refined using a riding model. Non-H atoms were refined with anisotropic displacement parameters. H-atom displacement parameters were set at 1.2 times the isotropic equivalent displacement parameters of the bonded atoms.

## Supplementary Material

Crystal structure: contains datablock(s) I. DOI: 10.1107/S2056989026007024/oi2040sup1.cif

Structure factors: contains datablock(s) I. DOI: 10.1107/S2056989026007024/oi2040Isup2.hkl

Fig. S1: IR spectrum of compound 1. DOI: 10.1107/S2056989026007024/oi2040sup3.jpg

Fig. S2: An SEM image (top) and XPS (bottom) of a sample of compound 1. DOI: 10.1107/S2056989026007024/oi2040sup4.jpg

Supporting information file. DOI: 10.1107/S2056989026007024/oi2040Isup5.mol

CCDC reference: 2570948

Additional supporting information:  crystallographic information; 3D view; checkCIF report

## Figures and Tables

**Table 1:** Experimental details

Crystal data	
Chemical formula	[Ti(C_6_F_5_O)_4_(C_4_H_8_O)_2_]
*M*_r_	924.35
Crystal system, space group	Monoclinic, *P*2_1_/*n*
Temperature (K)	100
*a*, *b*, *c* (Å)	10.7678 (3), 18.6224 (5), 17.1245 (5)
β (°)	90.204 (2)
*V* (Å^3^)	3433.82 (17)
*Z*	4
Radiation type	Mo *K*α
μ (mm^−1^)	0.40
Crystal size (mm)	0.14 × 0.14 × 0.09

Data collection	
Diffractometer	Bruker APEX CCD
Absorption correction	Multi-scan (*SADABS2016*; Krause *et al.*, 2015 ▸)
*T*_min_, *T*_max_	0.623, 0.695
No. of measured, independent and observed [*I* > 2σ(*I*)] reflections	41654, 6043, 4550
*R*_int_	0.068
(sin θ/λ)_max_ (Å^−1^)	0.595

Refinement	
*R*[*F*^2^ > 2σ(*F*^2^)], *wR*(*F*^2^), *S*	0.039, 0.097, 1.00
No. of reflections	6043
No. of parameters	532
H-atom treatment	H-atom parameters constrained
Δρ_max_, Δρ_min_ (e Å^−3^)	0.34, −0.46

Computer programs: *APEX3* (Bruker, 2007 ▸), *SAINT* (Bruker, 2007 ▸), *SHELXT* (Sheldrick, 2015 ▸), *SHELXL2025* (Sheldrick, 2025 ▸) and *OLEX2* (Dolomanov *et al.*, 2009 ▸).

## supplementary crystallographic information

### Tetrakis(pentafluorophenoxido-κO)bis(tetrahydrofuran-κO)titanium(IV), [Ti(C6F5O)4(C4H8O)2] Crystal data

**Table:**

[Ti(C_6_F_5_O)_4_(C_4_H_8_O)_2_]	*F*(000) = 1832
*M**_r_* = 924.35	*D*_x_ = 1.788 Mg m^−^^3^
Monoclinic, *P*2_1_/*n*	Mo *K*α radiation, λ = 0.71073 Å
*a* = 10.7678 (3) Å	Cell parameters from 8410 reflections
*b* = 18.6224 (5) Å	θ = 2.4–30.4°
*c* = 17.1245 (5) Å	µ = 0.40 mm^−^^1^
β = 90.204 (2)°	*T* = 100 K
*V* = 3433.82 (17) Å^3^	Block, orange
*Z* = 4	0.14 × 0.14 × 0.09 mm

### Tetrakis(pentafluorophenoxido-κO)bis(tetrahydrofuran-κO)titanium(IV), [Ti(C6F5O)4(C4H8O)2] Data collection

**Table:**

Bruker APEX CCD diffractometer	4550 reflections with *I* > 2σ(*I*)
φ and ω scans	*R*_int_ = 0.068
Absorption correction: multi-scan (SADABS2016; Krause *et al.*, 2015)	θ_max_ = 25.0°, θ_min_ = 2.2°
*T*_min_ = 0.623, *T*_max_ = 0.695	*h* = −12→12
41654 measured reflections	*k* = −22→22
6043 independent reflections	*l* = −19→20

### Tetrakis(pentafluorophenoxido-κO)bis(tetrahydrofuran-κO)titanium(IV), [Ti(C6F5O)4(C4H8O)2] Refinement

**Table:**

Refinement on *F*^2^	0 restraints
Least-squares matrix: full	Hydrogen site location: inferred from neighbouring sites
*R*[*F*^2^ > 2σ(*F*^2^)] = 0.039	H-atom parameters constrained
*wR*(*F*^2^) = 0.097	*w* = 1/[σ^2^(*F*_o_^2^) + (0.0415*P*)^2^ + 2.4623*P*] where *P* = (*F*_o_^2^ + 2*F*_c_^2^)/3
*S* = 1.00	(Δ/σ)_max_ = 0.001
6043 reflections	Δρ_max_ = 0.34 e Å^−^^3^
532 parameters	Δρ_min_ = −0.46 e Å^−^^3^

### Tetrakis(pentafluorophenoxido-κO)bis(tetrahydrofuran-κO)titanium(IV), [Ti(C6F5O)4(C4H8O)2] Special details

**Table:**

Geometry. All esds (except the esd in the dihedral angle between two l.s. planes) are estimated using the full covariance matrix. The cell esds are taken into account individually in the estimation of esds in distances, angles and torsion angles; correlations between esds in cell parameters are only used when they are defined by crystal symmetry. An approximate (isotropic) treatment of cell esds is used for estimating esds involving l.s. planes.

### Tetrakis(pentafluorophenoxido-κO)bis(tetrahydrofuran-κO)titanium(IV), [Ti(C6F5O)4(C4H8O)2] Fractional atomic coordinates and isotropic or equivalent isotropic displacement parameters (Å^2^)

**Table:**

	*x*	*y*	*z*	*U*_iso_*/*U*_eq_	
Ti1	0.54379 (4)	0.21700 (2)	0.65158 (3)	0.01836 (13)	
F2	0.48061 (15)	0.07750 (8)	0.46061 (9)	0.0275 (4)	
F3	0.46785 (16)	0.08295 (9)	0.30352 (10)	0.0361 (4)	
F4	0.52382 (17)	0.20695 (10)	0.22618 (9)	0.0392 (4)	
F5	0.58924 (15)	0.32564 (9)	0.30822 (10)	0.0330 (4)	
F6	0.60174 (15)	0.32125 (8)	0.46622 (9)	0.0285 (4)	
F8	0.30266 (16)	0.27450 (9)	0.50221 (10)	0.0389 (5)	
F9	0.27079 (18)	0.39576 (11)	0.41814 (11)	0.0532 (6)	
F10	0.33334 (18)	0.52580 (10)	0.48005 (12)	0.0534 (6)	
F11	0.42690 (17)	0.53281 (9)	0.62739 (12)	0.0439 (5)	
F12	0.45684 (16)	0.41232 (8)	0.71255 (10)	0.0311 (4)	
F14	0.82314 (16)	0.23242 (8)	0.53812 (11)	0.0375 (4)	
F15	1.02058 (17)	0.30959 (10)	0.48959 (12)	0.0504 (5)	
F16	1.06352 (16)	0.44323 (9)	0.54877 (11)	0.0417 (5)	
F17	0.90700 (15)	0.49807 (8)	0.65763 (10)	0.0350 (4)	
F18	0.71103 (16)	0.42083 (8)	0.70748 (10)	0.0344 (4)	
F19	0.31651 (14)	0.25946 (9)	0.80634 (10)	0.0299 (4)	
F20	0.29102 (14)	0.36793 (8)	0.91024 (9)	0.0286 (4)	
F21	0.49437 (15)	0.42573 (8)	0.98074 (9)	0.0289 (4)	
F22	0.72545 (14)	0.37964 (8)	0.93880 (9)	0.0283 (4)	
F23	0.75251 (14)	0.27640 (8)	0.83031 (9)	0.0265 (4)	
O1	0.54289 (18)	0.19743 (9)	0.54538 (11)	0.0239 (4)	
O2	0.40712 (17)	0.27960 (9)	0.64940 (11)	0.0240 (4)	
O3	0.66287 (17)	0.28759 (9)	0.64923 (11)	0.0254 (4)	
O4	0.54803 (16)	0.21227 (9)	0.76279 (10)	0.0210 (4)	
O5	0.67766 (17)	0.13425 (9)	0.66137 (10)	0.0217 (4)	
O6	0.41500 (17)	0.13131 (9)	0.66251 (11)	0.0232 (4)	
C1	0.5398 (2)	0.19959 (13)	0.46761 (15)	0.0179 (6)	
C2	0.5075 (2)	0.13973 (14)	0.42396 (16)	0.0209 (6)	
C3	0.5019 (2)	0.14218 (15)	0.34369 (17)	0.0241 (6)	
C4	0.5293 (3)	0.20430 (15)	0.30475 (16)	0.0244 (6)	
C5	0.5616 (2)	0.26442 (14)	0.34650 (16)	0.0216 (6)	
C6	0.5680 (2)	0.26185 (13)	0.42646 (16)	0.0202 (6)	
C7	0.3861 (2)	0.33975 (14)	0.60855 (16)	0.0233 (6)	
C8	0.3355 (3)	0.33817 (16)	0.53372 (18)	0.0293 (7)	
C9	0.3184 (3)	0.39969 (18)	0.49046 (18)	0.0361 (8)	
C10	0.3496 (3)	0.46504 (17)	0.52192 (19)	0.0359 (8)	
C11	0.3969 (3)	0.46866 (15)	0.59653 (19)	0.0315 (7)	
C12	0.4132 (3)	0.40705 (15)	0.63897 (17)	0.0256 (6)	
C13	0.7608 (2)	0.32487 (13)	0.62373 (15)	0.0203 (6)	
C14	0.8424 (3)	0.29829 (14)	0.56797 (17)	0.0257 (6)	
C15	0.9432 (3)	0.33737 (16)	0.54330 (17)	0.0299 (7)	
C16	0.9649 (3)	0.40483 (15)	0.57279 (17)	0.0270 (7)	
C17	0.8863 (3)	0.43218 (14)	0.62814 (17)	0.0243 (6)	
C18	0.7862 (2)	0.39280 (14)	0.65310 (16)	0.0218 (6)	
C19	0.5350 (2)	0.26492 (13)	0.81489 (15)	0.0174 (6)	
C20	0.6369 (2)	0.29737 (13)	0.85021 (16)	0.0193 (6)	
C21	0.6242 (2)	0.35047 (13)	0.90531 (16)	0.0205 (6)	
C22	0.5080 (3)	0.37395 (13)	0.92656 (15)	0.0207 (6)	
C23	0.4055 (2)	0.34380 (14)	0.89140 (16)	0.0204 (6)	
C24	0.4187 (2)	0.28991 (14)	0.83767 (15)	0.0195 (6)	
C25	0.7764 (3)	0.13369 (16)	0.72070 (17)	0.0303 (7)	
H25A	0.746264	0.112362	0.770084	0.036*	
H25B	0.806424	0.182994	0.731279	0.036*	
C26	0.8780 (3)	0.08829 (16)	0.68563 (19)	0.0332 (7)	
H26A	0.927828	0.064229	0.726705	0.040*	
H26B	0.933751	0.117468	0.652470	0.040*	
C27	0.8055 (3)	0.03401 (14)	0.63716 (17)	0.0293 (7)	
H27A	0.858044	0.012813	0.595813	0.035*	
H27B	0.772060	−0.004972	0.670294	0.035*	
C28	0.7021 (3)	0.07884 (14)	0.60249 (16)	0.0247 (6)	
H28A	0.728349	0.100807	0.552551	0.030*	
H28B	0.627176	0.049252	0.593037	0.030*	
C29	0.4092 (3)	0.07952 (15)	0.72628 (18)	0.0347 (8)	
H29A	0.406771	0.104471	0.777298	0.042*	
H29B	0.482501	0.047409	0.725375	0.042*	
C30	0.2905 (3)	0.03692 (16)	0.7130 (2)	0.0380 (8)	
H30A	0.304888	−0.015034	0.721297	0.046*	
H30B	0.223958	0.053268	0.748501	0.046*	
C31	0.2572 (3)	0.05210 (18)	0.6292 (2)	0.0456 (9)	
H31A	0.301009	0.018998	0.593386	0.055*	
H31B	0.166589	0.047790	0.620419	0.055*	
C32	0.3000 (3)	0.12791 (16)	0.61809 (19)	0.0322 (7)	
H32A	0.314747	0.138376	0.562205	0.039*	
H32B	0.238228	0.162378	0.638617	0.039*	

### Tetrakis(pentafluorophenoxido-κO)bis(tetrahydrofuran-κO)titanium(IV), [Ti(C6F5O)4(C4H8O)2] Atomic displacement parameters (Å^2^)

**Table:**

	*U*^11^	*U*^22^	*U*^33^	*U*^12^	*U*^13^	*U*^23^
Ti1	0.0195 (3)	0.0161 (2)	0.0194 (3)	−0.00047 (19)	0.0011 (2)	−0.0020 (2)
F2	0.0306 (9)	0.0192 (8)	0.0327 (10)	−0.0024 (7)	−0.0023 (7)	0.0027 (7)
F3	0.0415 (11)	0.0354 (9)	0.0313 (10)	−0.0037 (8)	−0.0082 (8)	−0.0142 (8)
F4	0.0424 (11)	0.0569 (12)	0.0184 (10)	−0.0050 (9)	−0.0027 (8)	0.0001 (8)
F5	0.0330 (10)	0.0343 (9)	0.0316 (10)	−0.0014 (8)	0.0022 (8)	0.0128 (8)
F6	0.0367 (10)	0.0187 (8)	0.0303 (10)	−0.0020 (7)	0.0008 (7)	−0.0030 (7)
F8	0.0360 (10)	0.0458 (11)	0.0348 (11)	0.0117 (8)	−0.0100 (8)	−0.0138 (9)
F9	0.0499 (13)	0.0814 (15)	0.0280 (11)	0.0230 (11)	−0.0068 (9)	0.0092 (10)
F10	0.0467 (12)	0.0537 (12)	0.0598 (14)	0.0116 (10)	0.0023 (10)	0.0352 (10)
F11	0.0465 (12)	0.0229 (9)	0.0623 (13)	0.0021 (8)	0.0018 (10)	0.0067 (8)
F12	0.0384 (10)	0.0241 (8)	0.0307 (10)	0.0034 (7)	−0.0037 (8)	−0.0005 (7)
F14	0.0363 (10)	0.0299 (9)	0.0465 (12)	−0.0084 (8)	0.0153 (8)	−0.0211 (8)
F15	0.0387 (11)	0.0573 (12)	0.0554 (13)	−0.0122 (9)	0.0263 (10)	−0.0242 (10)
F16	0.0271 (10)	0.0412 (10)	0.0568 (13)	−0.0143 (8)	0.0083 (9)	0.0010 (9)
F17	0.0348 (10)	0.0201 (8)	0.0502 (12)	−0.0078 (7)	−0.0045 (8)	−0.0067 (8)
F18	0.0341 (10)	0.0291 (9)	0.0399 (11)	−0.0026 (7)	0.0113 (8)	−0.0162 (8)
F19	0.0159 (8)	0.0402 (9)	0.0338 (10)	−0.0069 (7)	0.0026 (7)	−0.0108 (8)
F20	0.0178 (8)	0.0334 (9)	0.0348 (10)	0.0041 (7)	0.0089 (7)	−0.0058 (7)
F21	0.0349 (10)	0.0222 (8)	0.0296 (10)	−0.0008 (7)	0.0060 (7)	−0.0105 (7)
F22	0.0232 (9)	0.0294 (8)	0.0322 (10)	−0.0030 (7)	−0.0082 (7)	−0.0069 (7)
F23	0.0149 (8)	0.0322 (8)	0.0322 (10)	0.0025 (7)	−0.0008 (7)	−0.0070 (7)
O1	0.0301 (11)	0.0221 (9)	0.0194 (11)	0.0002 (8)	0.0005 (8)	−0.0005 (8)
O2	0.0245 (11)	0.0215 (9)	0.0260 (11)	0.0037 (8)	0.0010 (8)	0.0003 (8)
O3	0.0251 (11)	0.0225 (10)	0.0287 (12)	−0.0041 (8)	0.0059 (9)	−0.0035 (8)
O4	0.0208 (10)	0.0203 (9)	0.0219 (11)	0.0003 (8)	0.0022 (8)	−0.0045 (8)
O5	0.0220 (10)	0.0222 (9)	0.0210 (11)	0.0030 (8)	−0.0011 (8)	−0.0050 (8)
O6	0.0241 (10)	0.0219 (9)	0.0236 (11)	−0.0040 (8)	−0.0036 (8)	0.0027 (8)
C1	0.0176 (14)	0.0216 (13)	0.0145 (14)	0.0029 (11)	−0.0013 (11)	−0.0001 (11)
C2	0.0179 (14)	0.0209 (13)	0.0240 (16)	−0.0005 (11)	−0.0008 (11)	−0.0010 (12)
C3	0.0162 (14)	0.0291 (15)	0.0271 (17)	0.0000 (12)	−0.0039 (12)	−0.0091 (13)
C4	0.0176 (14)	0.0400 (17)	0.0157 (15)	0.0009 (12)	−0.0016 (11)	−0.0015 (13)
C5	0.0157 (14)	0.0265 (14)	0.0225 (16)	−0.0015 (11)	0.0013 (11)	0.0067 (12)
C6	0.0162 (14)	0.0207 (13)	0.0235 (16)	0.0024 (11)	−0.0013 (11)	−0.0050 (11)
C7	0.0193 (14)	0.0252 (14)	0.0253 (16)	0.0067 (12)	0.0022 (12)	0.0021 (12)
C8	0.0220 (15)	0.0346 (16)	0.0314 (18)	0.0092 (13)	0.0027 (13)	−0.0060 (14)
C9	0.0294 (17)	0.054 (2)	0.0251 (18)	0.0159 (15)	−0.0012 (14)	0.0071 (15)
C10	0.0275 (17)	0.0403 (18)	0.040 (2)	0.0107 (14)	0.0079 (15)	0.0216 (16)
C11	0.0272 (17)	0.0250 (15)	0.042 (2)	0.0025 (13)	0.0059 (14)	0.0064 (14)
C12	0.0218 (15)	0.0297 (15)	0.0253 (16)	0.0031 (12)	0.0023 (12)	0.0030 (13)
C13	0.0205 (14)	0.0216 (13)	0.0187 (15)	−0.0029 (11)	0.0007 (11)	−0.0008 (11)
C14	0.0284 (16)	0.0200 (14)	0.0287 (17)	−0.0033 (12)	−0.0006 (13)	−0.0064 (12)
C15	0.0260 (16)	0.0349 (16)	0.0288 (18)	−0.0008 (13)	0.0075 (13)	−0.0072 (13)
C16	0.0194 (15)	0.0304 (15)	0.0313 (18)	−0.0059 (12)	0.0016 (13)	0.0017 (13)
C17	0.0269 (16)	0.0168 (13)	0.0293 (17)	−0.0014 (12)	−0.0086 (13)	−0.0009 (12)
C18	0.0202 (14)	0.0221 (14)	0.0232 (16)	0.0029 (11)	0.0013 (12)	−0.0047 (12)
C19	0.0200 (14)	0.0166 (12)	0.0156 (14)	−0.0003 (11)	0.0019 (11)	0.0009 (11)
C20	0.0128 (13)	0.0220 (13)	0.0231 (15)	0.0032 (11)	0.0039 (11)	0.0018 (11)
C21	0.0181 (14)	0.0208 (13)	0.0226 (15)	−0.0049 (11)	−0.0040 (11)	0.0003 (11)
C22	0.0250 (15)	0.0178 (13)	0.0192 (15)	0.0005 (11)	0.0039 (12)	−0.0012 (11)
C23	0.0151 (14)	0.0241 (14)	0.0219 (15)	0.0024 (11)	0.0068 (11)	0.0031 (11)
C24	0.0174 (14)	0.0243 (13)	0.0170 (14)	−0.0045 (11)	0.0008 (11)	0.0016 (11)
C25	0.0263 (16)	0.0374 (16)	0.0273 (17)	0.0060 (13)	−0.0067 (13)	−0.0071 (13)
C26	0.0270 (17)	0.0330 (16)	0.0396 (19)	0.0090 (13)	−0.0019 (14)	−0.0047 (14)
C27	0.0400 (18)	0.0218 (14)	0.0261 (17)	0.0073 (13)	0.0064 (14)	0.0023 (12)
C28	0.0321 (17)	0.0191 (13)	0.0227 (16)	0.0017 (12)	0.0054 (12)	−0.0063 (12)
C29	0.046 (2)	0.0271 (15)	0.0309 (18)	−0.0110 (14)	−0.0019 (15)	0.0081 (13)
C30	0.0323 (18)	0.0322 (16)	0.049 (2)	−0.0087 (14)	0.0087 (16)	0.0071 (15)
C31	0.037 (2)	0.0425 (19)	0.058 (2)	−0.0156 (16)	−0.0047 (17)	−0.0011 (17)
C32	0.0236 (16)	0.0362 (17)	0.0369 (19)	−0.0074 (13)	−0.0096 (14)	0.0041 (14)

### Tetrakis(pentafluorophenoxido-κO)bis(tetrahydrofuran-κO)titanium(IV), [Ti(C6F5O)4(C4H8O)2] Geometric parameters (Å, º)

**Table:**

Ti1—O1	1.8548 (19)	C7—C8	1.391 (4)
Ti1—O2	1.8777 (18)	C7—C12	1.388 (4)
Ti1—O3	1.8370 (18)	C8—C9	1.376 (4)
Ti1—O4	1.9068 (19)	C9—C10	1.372 (5)
Ti1—O5	2.1163 (18)	C10—C11	1.375 (4)
Ti1—O6	2.1227 (18)	C11—C12	1.369 (4)
F2—C2	1.350 (3)	C13—C14	1.391 (4)
F3—C3	1.350 (3)	C13—C18	1.388 (4)
F4—C4	1.347 (3)	C14—C15	1.375 (4)
F5—C5	1.349 (3)	C15—C16	1.374 (4)
F6—C6	1.348 (3)	C16—C17	1.371 (4)
F8—C8	1.349 (3)	C17—C18	1.374 (4)
F9—C9	1.341 (3)	C19—C20	1.390 (4)
F10—C10	1.351 (3)	C19—C24	1.392 (4)
F11—C11	1.345 (3)	C20—C21	1.374 (4)
F12—C12	1.347 (3)	C21—C22	1.376 (4)
F14—C14	1.345 (3)	C22—C23	1.375 (4)
F15—C15	1.347 (3)	C23—C24	1.369 (4)
F16—C16	1.346 (3)	C25—H25A	0.9900
F17—C17	1.345 (3)	C25—H25B	0.9900
F18—C18	1.341 (3)	C25—C26	1.509 (4)
F19—C24	1.348 (3)	C26—H26A	0.9900
F20—C23	1.352 (3)	C26—H26B	0.9900
F21—C22	1.347 (3)	C26—C27	1.522 (4)
F22—C21	1.344 (3)	C27—H27A	0.9900
F23—C20	1.350 (3)	C27—H27B	0.9900
O1—C1	1.333 (3)	C27—C28	1.511 (4)
O2—C7	1.340 (3)	C28—H28A	0.9900
O3—C13	1.337 (3)	C28—H28B	0.9900
O4—C19	1.333 (3)	C29—H29A	0.9900
O5—C25	1.468 (3)	C29—H29B	0.9900
O5—C28	1.467 (3)	C29—C30	1.521 (4)
O6—C29	1.458 (3)	C30—H30A	0.9900
O6—C32	1.452 (3)	C30—H30B	0.9900
C1—C2	1.386 (4)	C30—C31	1.505 (5)
C1—C6	1.391 (4)	C31—H31A	0.9900
C2—C3	1.376 (4)	C31—H31B	0.9900
C3—C4	1.368 (4)	C31—C32	1.497 (4)
C4—C5	1.373 (4)	C32—H32A	0.9900
C5—C6	1.371 (4)	C32—H32B	0.9900
			
O1—Ti1—O2	95.80 (8)	F17—C17—C16	119.8 (2)
O1—Ti1—O4	165.98 (8)	F17—C17—C18	119.9 (3)
O1—Ti1—O5	86.32 (8)	C18—C17—C16	120.3 (2)
O1—Ti1—O6	86.42 (8)	F18—C18—C13	119.2 (2)
O2—Ti1—O4	93.70 (8)	F18—C18—C17	119.0 (2)
O2—Ti1—O5	170.82 (8)	C17—C18—C13	121.8 (2)
O2—Ti1—O6	87.49 (8)	O4—C19—C20	121.7 (2)
O3—Ti1—O1	96.91 (8)	O4—C19—C24	122.0 (2)
O3—Ti1—O2	95.87 (8)	C20—C19—C24	116.2 (2)
O3—Ti1—O4	92.33 (8)	F23—C20—C19	119.5 (2)
O3—Ti1—O5	92.73 (8)	F23—C20—C21	118.4 (2)
O3—Ti1—O6	174.99 (8)	C21—C20—C19	122.1 (2)
O4—Ti1—O5	82.73 (7)	F22—C21—C20	120.1 (2)
O4—Ti1—O6	83.72 (7)	F22—C21—C22	119.7 (2)
O5—Ti1—O6	83.72 (7)	C20—C21—C22	120.2 (2)
C1—O1—Ti1	166.88 (17)	F21—C22—C21	120.7 (2)
C7—O2—Ti1	131.35 (17)	F21—C22—C23	120.3 (2)
C13—O3—Ti1	158.45 (17)	C23—C22—C21	118.9 (2)
C19—O4—Ti1	129.21 (16)	F20—C23—C22	119.4 (2)
C25—O5—Ti1	123.46 (15)	F20—C23—C24	120.1 (2)
C25—O5—C28	109.84 (19)	C24—C23—C22	120.5 (2)
C28—O5—Ti1	125.56 (15)	F19—C24—C19	118.8 (2)
C29—O6—Ti1	126.34 (16)	F19—C24—C23	119.3 (2)
C32—O6—Ti1	122.87 (15)	C23—C24—C19	122.0 (2)
C32—O6—C29	109.0 (2)	O5—C25—H25A	110.8
O1—C1—C2	121.3 (2)	O5—C25—H25B	110.8
O1—C1—C6	121.8 (2)	O5—C25—C26	104.7 (2)
C2—C1—C6	116.9 (2)	H25A—C25—H25B	108.9
F2—C2—C1	119.6 (2)	C26—C25—H25A	110.8
F2—C2—C3	119.0 (2)	C26—C25—H25B	110.8
C3—C2—C1	121.5 (2)	C25—C26—H26A	111.3
F3—C3—C2	119.5 (2)	C25—C26—H26B	111.3
F3—C3—C4	120.1 (3)	C25—C26—C27	102.6 (2)
C4—C3—C2	120.4 (2)	H26A—C26—H26B	109.2
F4—C4—C3	120.6 (2)	C27—C26—H26A	111.3
F4—C4—C5	120.0 (2)	C27—C26—H26B	111.3
C3—C4—C5	119.4 (3)	C26—C27—H27A	111.2
F5—C5—C4	119.5 (2)	C26—C27—H27B	111.2
F5—C5—C6	120.3 (2)	H27A—C27—H27B	109.1
C4—C5—C6	120.2 (2)	C28—C27—C26	102.9 (2)
F6—C6—C1	119.1 (2)	C28—C27—H27A	111.2
F6—C6—C5	119.2 (2)	C28—C27—H27B	111.2
C5—C6—C1	121.6 (2)	O5—C28—C27	104.6 (2)
O2—C7—C8	121.9 (2)	O5—C28—H28A	110.8
O2—C7—C12	121.6 (3)	O5—C28—H28B	110.8
C12—C7—C8	116.5 (3)	C27—C28—H28A	110.8
F8—C8—C7	119.2 (3)	C27—C28—H28B	110.8
F8—C8—C9	118.8 (3)	H28A—C28—H28B	108.9
C9—C8—C7	122.0 (3)	O6—C29—H29A	110.6
F9—C9—C8	120.1 (3)	O6—C29—H29B	110.6
F9—C9—C10	120.2 (3)	O6—C29—C30	105.7 (2)
C10—C9—C8	119.6 (3)	H29A—C29—H29B	108.7
F10—C10—C9	120.2 (3)	C30—C29—H29A	110.6
F10—C10—C11	119.9 (3)	C30—C29—H29B	110.6
C9—C10—C11	119.8 (3)	C29—C30—H30A	111.0
F11—C11—C10	119.7 (3)	C29—C30—H30B	111.0
F11—C11—C12	120.4 (3)	H30A—C30—H30B	109.0
C12—C11—C10	119.9 (3)	C31—C30—C29	104.0 (2)
F12—C12—C7	119.3 (2)	C31—C30—H30A	111.0
F12—C12—C11	118.6 (3)	C31—C30—H30B	111.0
C11—C12—C7	122.1 (3)	C30—C31—H31A	111.2
O3—C13—C14	122.7 (2)	C30—C31—H31B	111.2
O3—C13—C18	120.6 (2)	H31A—C31—H31B	109.1
C14—C13—C18	116.7 (2)	C32—C31—C30	103.0 (3)
F14—C14—C13	119.3 (2)	C32—C31—H31A	111.2
F14—C14—C15	119.1 (3)	C32—C31—H31B	111.2
C15—C14—C13	121.6 (2)	O6—C32—C31	103.7 (2)
F15—C15—C14	119.8 (3)	O6—C32—H32A	111.0
F15—C15—C16	119.8 (3)	O6—C32—H32B	111.0
C14—C15—C16	120.3 (3)	C31—C32—H32A	111.0
F16—C16—C15	120.5 (3)	C31—C32—H32B	111.0
F16—C16—C17	120.2 (3)	H32A—C32—H32B	109.0
C17—C16—C15	119.3 (3)		
			
Ti1—O1—C1—C2	−159.0 (7)	O4—C19—C20—C21	−178.4 (2)
Ti1—O1—C1—C6	20.6 (9)	O4—C19—C24—F19	0.5 (4)
Ti1—O2—C7—C8	86.4 (3)	O4—C19—C24—C23	179.7 (2)
Ti1—O2—C7—C12	−93.8 (3)	O5—Ti1—O1—C1	−141.9 (8)
Ti1—O3—C13—C14	−14.4 (7)	O5—Ti1—O3—C13	61.0 (5)
Ti1—O3—C13—C18	166.3 (4)	O5—C25—C26—C27	−32.4 (3)
Ti1—O4—C19—C20	−98.6 (3)	O6—Ti1—O1—C1	134.1 (8)
Ti1—O4—C19—C24	82.2 (3)	O6—Ti1—O2—C7	−142.4 (2)
Ti1—O5—C25—C26	−155.01 (18)	O6—C29—C30—C31	17.0 (3)
Ti1—O5—C28—C27	179.54 (16)	C1—C2—C3—F3	−178.8 (2)
Ti1—O6—C29—C30	171.78 (18)	C1—C2—C3—C4	0.4 (4)
Ti1—O6—C32—C31	166.3 (2)	C2—C1—C6—F6	−179.0 (2)
F2—C2—C3—F3	1.1 (4)	C2—C1—C6—C5	1.3 (4)
F2—C2—C3—C4	−179.6 (2)	C2—C3—C4—F4	−179.9 (2)
F3—C3—C4—F4	−0.7 (4)	C2—C3—C4—C5	−0.4 (4)
F3—C3—C4—C5	178.9 (2)	C3—C4—C5—F5	179.7 (2)
F4—C4—C5—F5	−0.8 (4)	C3—C4—C5—C6	0.9 (4)
F4—C4—C5—C6	−179.6 (2)	C4—C5—C6—F6	179.0 (2)
F5—C5—C6—F6	0.2 (4)	C4—C5—C6—C1	−1.3 (4)
F5—C5—C6—C1	179.9 (2)	C6—C1—C2—F2	179.2 (2)
F8—C8—C9—F9	−0.6 (4)	C6—C1—C2—C3	−0.9 (4)
F8—C8—C9—C10	179.1 (3)	C7—C8—C9—F9	179.2 (3)
F9—C9—C10—F10	−0.4 (5)	C7—C8—C9—C10	−1.2 (5)
F9—C9—C10—C11	179.2 (3)	C8—C7—C12—F12	176.9 (2)
F10—C10—C11—F11	−0.1 (4)	C8—C7—C12—C11	−3.0 (4)
F10—C10—C11—C12	179.9 (3)	C8—C9—C10—F10	180.0 (3)
F11—C11—C12—F12	1.6 (4)	C8—C9—C10—C11	−0.5 (5)
F11—C11—C12—C7	−178.5 (3)	C9—C10—C11—F11	−179.7 (3)
F14—C14—C15—F15	0.4 (4)	C9—C10—C11—C12	0.4 (5)
F14—C14—C15—C16	179.9 (3)	C10—C11—C12—F12	−178.4 (3)
F15—C15—C16—F16	−0.4 (4)	C10—C11—C12—C7	1.5 (4)
F15—C15—C16—C17	−179.2 (3)	C12—C7—C8—F8	−177.4 (2)
F16—C16—C17—F17	1.0 (4)	C12—C7—C8—C9	2.8 (4)
F16—C16—C17—C18	−179.5 (3)	C13—C14—C15—F15	179.5 (3)
F17—C17—C18—F18	−0.7 (4)	C13—C14—C15—C16	−0.9 (5)
F17—C17—C18—C13	179.4 (2)	C14—C13—C18—F18	−179.5 (2)
F20—C23—C24—F19	−2.6 (4)	C14—C13—C18—C17	0.4 (4)
F20—C23—C24—C19	178.1 (2)	C14—C15—C16—F16	180.0 (3)
F21—C22—C23—F20	1.7 (4)	C14—C15—C16—C17	1.2 (5)
F21—C22—C23—C24	−178.4 (2)	C15—C16—C17—F17	179.8 (3)
F22—C21—C22—F21	−0.2 (4)	C15—C16—C17—C18	−0.7 (4)
F22—C21—C22—C23	179.6 (2)	C16—C17—C18—F18	179.8 (2)
F23—C20—C21—F22	−0.9 (4)	C16—C17—C18—C13	−0.1 (4)
F23—C20—C21—C22	179.2 (2)	C18—C13—C14—F14	179.3 (2)
O1—Ti1—O2—C7	−56.3 (2)	C18—C13—C14—C15	0.1 (4)
O1—Ti1—O3—C13	−25.6 (5)	C19—C20—C21—F22	179.0 (2)
O1—C1—C2—F2	−1.2 (4)	C19—C20—C21—C22	−0.9 (4)
O1—C1—C2—C3	178.7 (2)	C20—C19—C24—F19	−178.8 (2)
O1—C1—C6—F6	1.3 (4)	C20—C19—C24—C23	0.5 (4)
O1—C1—C6—C5	−178.3 (2)	C20—C21—C22—F21	179.7 (2)
O2—Ti1—O1—C1	47.0 (8)	C20—C21—C22—C23	−0.5 (4)
O2—Ti1—O3—C13	−122.2 (5)	C21—C22—C23—F20	−178.1 (2)
O2—C7—C8—F8	2.3 (4)	C21—C22—C23—C24	1.8 (4)
O2—C7—C8—C9	−177.4 (3)	C22—C23—C24—F19	177.5 (2)
O2—C7—C12—F12	−2.9 (4)	C22—C23—C24—C19	−1.8 (4)
O2—C7—C12—C11	177.2 (3)	C24—C19—C20—F23	−179.3 (2)
O3—Ti1—O1—C1	−49.6 (8)	C24—C19—C20—C21	0.8 (4)
O3—Ti1—O2—C7	41.3 (2)	C25—O5—C28—C27	11.5 (3)
O3—C13—C14—F14	−0.1 (4)	C25—C26—C27—C28	39.3 (3)
O3—C13—C14—C15	−179.3 (3)	C26—C27—C28—O5	−31.4 (3)
O3—C13—C18—F18	−0.1 (4)	C28—O5—C25—C26	13.4 (3)
O3—C13—C18—C17	179.8 (3)	C29—O6—C32—C31	−28.2 (3)
O4—Ti1—O1—C1	179.5 (7)	C29—C30—C31—C32	−33.7 (3)
O4—Ti1—O2—C7	134.1 (2)	C30—C31—C32—O6	38.1 (3)
O4—Ti1—O3—C13	143.8 (5)	C32—O6—C29—C30	7.0 (3)
O4—C19—C20—F23	1.5 (4)		

### Tetrakis(pentafluorophenoxido-κO)bis(tetrahydrofuran-κO)titanium(IV), [Ti(C6F5O)4(C4H8O)2] Hydrogen-bond geometry (Å, º)

**Table:**

*D*—H···*A*	*D*—H	H···*A*	*D*···*A*	*D*—H···*A*
C25—H25*A*···F9^i^	0.99	2.55	3.426 (4)	147
C25—H25*B*···F23	0.99	2.50	3.264 (3)	134
C27—H27*A*···F21^ii^	0.99	2.62	3.580 (3)	162
C27—H27*B*···F18^ii^	0.99	2.51	3.399 (3)	149
C28—H28*A*···F20^iii^	0.99	2.60	3.572 (3)	168
C29—H29*B*···F3^iv^	0.99	2.53	3.342 (4)	139
C29—H29*B*···F17^ii^	0.99	2.50	3.184 (4)	126
C32—H32*A*···F22^v^	0.99	2.34	3.174 (3)	141

Symmetry codes: (i) *x*+1/2, −*y*+1/2, *z*+1/2; (ii) −*x*+3/2, *y*−1/2, −*z*+3/2; (iii) *x*+1/2, −*y*+1/2, *z*−1/2; (iv) −*x*+1, −*y*, −*z*+1; (v) *x*−1/2, −*y*+1/2, *z*−1/2.
